# TUNEL – an efficient prognosis predictor of salivary malignancies

**DOI:** 10.1038/sj.bjc.6603655

**Published:** 2007-02-27

**Authors:** O Ben-Izhak, Z Laster, S Araidy, R M Nagler

**Affiliations:** 1 Department of Pathology, Rambam Medical Center, Haifa, Israel; 2 Department of Oral and Maxillofacial Surgery, Poriya Hospital, Tiberias, Israel; 3 Surgery and Oral Biochemistry Laboratory, Department of Oral and Maxillofacial Surgery, Rambam Medical Center and Bruce Rappaport Faculty of Medicine, Technion-Israel Institute of Technology, Haifa, Israel

**Keywords:** TUNEL, prognosis, malignancies, salivary glands

## Abstract

Biological markers are necessary for predicting prognosis of salivary malignancies and better understanding the pathogenesis of salivary cancer. We analysed terminal deoxynucleotidyl transferase (TdT)-mediated biotinylated deoxyuridine-triphosphate (dUTP)-biotin nick-end labelling (TUNEL), p53 and Ki67 expression in 66 patients with malignant salivary tumours by immonohistochemistry, and correlated the data with survival, disease-free survival, tumour grade, stage, and local and distant metastasis. TUNEL efficiently predicted poor prognosis in salivary malignancies. The 5-year (5Y) survival probability dropped significantly with the level of TUNEL staining (from 83% in negatively stained tumours to 57 and 24% in TUNEL positively stained levels 1 and 2, respectively), (*P*=0.042). Extensive Ki67 staining (in addition to TUNEL) reduced the 5Y-survival rate even further and addition of positively stained p53 dropped the 5Y-survival rate to 0. The correlation rates between TUNEL and Ki67 was 58% (*P*=0.0001), and between TUNEL and p53 it was 50% (*P*=0.035). Concurrently, TUNEL correlated with metastasis, extracapsular spread, grade and stage. The correlation between TUNEL, p53 and Ki67 staining and survival probabilities, and the pathological grade, stage and metastasis spread of salivary malignancies makes this a highly effective tool in patient follow-up and prognosis.

Salivary gland tumours are rare tumours of the head and neck region (Eneroth, 1971; Eveson and Cawson, 1985). The diagnosis and treatment of these tumours represent a challenge for head and neck surgeons for several reasons: they occur relatively infrequently, are found in diverse sites and show a great deal of variation in histological characteristics, clinical appearance, pathogenesis and 5-year (5Y) survival probability (Spiro and Dubner, 1990). These tumours may arise within the major salivary glands (parotid, submandibular (Sm) and sublingual (Sl)) or in one of the 600–1000 minor salivary glands spread throughout the oral mucosa (Johns and Goldsmith, 1989a). Their morphologic diversity and relative rarity frequently make it difficult to perform comparative analysis of their biologic, pathologic and clinical characteristics, which could facilitate estimating prognosis and establishing a preferable therapeutic modality (Batsakis, 1980; Dardick and van Nostrand, 1987; Batsakis *et al*, 1989; Johns and Goldsmith, 1989b; Dardick, 1991).

It is generally recognised that the prognosis of patients with salivary gland tumours depends basically on the salivary gland and the site in which they occur, the histological type, the extension of the disease at the time of diagnosis and the completeness of the surgical resection and/or radiotherapy administered (Eveson and Cawson, 1985; O’Brien *et al*, 1986; Tran *et al*, 1986; Hermanek *et al*, 1997; Batsakis, 1999). It is also known that salivary gland tumours have diverse outcomes: some patients are considered ‘cured’ decades after surgery, whereas others experience early recurrence and some die of the disease. As the clinical course and final outcome of many patients with salivary gland tumours cannot be reliably predicted on the basis of pathological histomorphologic features, it is highly desirable to pinpoint new prognosis markers aimed at better characterizing tumour aggressiveness and identifying especially subgroups of patients at the highest risk of recurrence who would benefit from adjuvant therapy. It is reasonable to assume that further improvement of the survival rate may be related to a better understanding of the biological nature of the disease, which in turn may facilitate the development of more efficient tools for the detection and treatment of these tumours. A significant effort has been made in finding valuable tumour markers for salivary-gland cancer in the last decade (Nagler *et al*, 1999).

In the current study, immunohistological examination of the following tumour markers were performed: the apoptosis regulatory terminal deoxynucleotidyl transferase (TdT)-mediated biotinylated deoxyuridine-triphosphate (dUTP)-biotin nick-end labelling (TUNEL) technique (for apoptosis rate assessment) and the carcinogenesis *vs* apoptosis-related p53 and the cell proliferation-rate-marker Ki67. We determined the percentage of apoptotic cells labelled by the well-established TUNEL method, which enables the visualization of apoptotic cells using an *in situ* end-labelling technique that labels DNA breaks in apoptotic cells (Ben-Sasson *et al*, 1995). Mutation in the p53 gene, which results in encoded non-functional protein is considered as the most common genetic event in human cancer. It has been suggested that mutated p53 may lead to carcinogenesis, as the wild-type p53 contributes to tumour suppression through at least two mechanisms in response to DNA damage, arrest of cell proliferation and induction of apoptosis (Attardi *et al*, 1996; Ravi *et al*, 1999; Xie *et al*, 1999).

It is unfortunate that only limited research has been devoted to examining these markers in salivary tumours, and that the TUNEL method has been only scarcely utilised. Moreover, all these markers have never been examined concomitantly or in relation to other clinical carcinogenic characteristics and in various types of malignant salivary tumours. In any case, given the mentioned variability and complexity of these tumours, a better understanding of their basic biology is required to define relevant targets for the development of novel therapeutic approaches. Thus, the purpose of the current study was to analyse all these and, in a relatively large cohort of patients, to correlate the obtained data with the survival and disease-free-survival (DFS) rates, and with other currently available clinical tools for prognosis prediction.

## MATERIALS AND METHODS

### Patients and experimental design

A total of 66 patients with a mean age of 60±16 years (range of 15–90 years) and a gender distribution of 41 (62%) males and 25 (38%) females who were diagnosed with various malignant salivary tumours were enrolled in the current study. Data concerning clinical tumour characteristics were collected and analysed: type of tumour, salivary gland involved (parotid, Sm, Sl or minor salivary glands), stage of disease, size (T), neck metastasis (N), distant metastasis (M) and the existence/absence of extracapsular spread of these neck metastases. The level of TUNEL staining of the tumours was correlated with all these clinical data as well as with patient survival and DFS probabilities. TUNEL-staining rate was also correlated with the staining rates of p53 and Ki67.

### Pathological study

All specimens were formalin-fixed and paraffin-embedded following surgical harvesting. Shortly before the immunological evaluation, serial sections (4 *μ*m in thickness) were prepared for haematoxylin and eosin staining, p53 and Ki67 immunostaining and for the TUNEL staining.

### TUNEL staining

Sections were stained by the *in situ* death-detection POD kit (Roche Diagnostic GmbH, Mannheim, Germany), according to the manufacturer's instructions. Briefly, after deparaffinisation and rehydration, sections were incubated with proteinase K (20 *μ*m ml^−1^ in 10 mM Tris–HCL, pH 7.4) for 30 min at 37°C. Slides were rinsed with PBS and incubated with 3% H_2_O_2_ in methanol for 10 min at room temperature to block endogenous peroxidase activity, followed by PBS washing and incubation in 0.1% Triton X-100 in 0.1% sodium citrate for 2 min on ice (4°C). Sections were incubated with a mixture of TdT solution and fluorescein isothiocyanate dUTP solution in a humidified chamber at 37°C for 60 min. This was followed by washings with PBS and incubation with antifluorescein antibody Fab fragments conjugated with horseradish peroxidase in a humidified chamber at 37°C for 30 min. After washing with PBS, aminoethyl carbazole solution was applied, followed by light counterstain with haematoxylin. Paraffin-embedded sections of normal tonsils were used as positive control. Negative control was obtained by replacing the TdT solution with distilled water. The presence of clear nuclear staining was indicative of apoptotic cells. At least 1000 tumour cell nuclei were examined in the most evenly and distinctly labelled areas. The number of TUNEL-positive tumour cell nuclei was counted and the apoptotic index was the percentage of apoptotic cells in the tumour. The apoptotic indices were classified into three groups: 3% (2).

### P53 and Ki67 staining

Four-micrometre paraffin-embedded sections were dewaxed and rehydrated. Endogenous peroxidase was blocked by incubation with 3% H_2_O_2_ in methanol for 10 min. Nonspecific binding was blocked by incubation in 10% normal serum for 20 min. Sections were heated in a microwave oven at 800 W for 15 min in 10 mM citrate buffer, pH 6. The primary antibodies used in the Ki67 study were anti-Ki67, clone MIB-1, diluted 1 : 40 (Zymed Laboratories. San Francisco, CA, USA). The antibodies used in the p53 study were anti-p53 (clone BP 53.12; 1 : 100, from Zymed Laboratories).

Slides were incubated with the antibodies and antiserum for 60 min at room temperature, followed by the application of the streptavidin–biotin complex method (histostain plus; Zymed Laboratories). Colour development was performed with aminoethyl cabazole followed by light haematoxylin counterstaining. Positive controls were run in parallel: colonic tumour, known to show strong p53, at least 500 tumour cells were counted in the areas with strongest staining. Positive staining for Ki67 was defined as level 1 (1–19%), level 2 (20–39%) or level 3 (40–60%). Positive staining for p53 was considered when more than 10% of tumour cells showed strong nuclear staining: staining of 10–30% of cells was classified as +1; and staining of >30% of cells was classified as +2.

### Statistical analysis

For categorical variables, frequencies and percentages were calculated. Distributions for categorical variables were compared and analysed by the Fisher–Irwin exact test (small sample). The Kaplan–Meier estimate was used to calculate the probability of survival and DFS rates as a function of time. The log rank test was used to compare the survival curves. The correlations between pairs of parameters were analysed by Pearson's correlation.

## RESULTS

### Tumour characteristics and survival rates

The 66 analysed malignant tumours included the following subgoups: mucoepidermoid (*n*=15), adeno carcinoma (*n*=12), squamous cell carcinoma (*n*=10), acinic cell carcinoma (*n*=9), adenoid cystic carcinoma (*n*=6), polymorphous low-grade adeno carcinoma (*n*=5), carcinoma ex mix tumour (*n*=2), salivary duct and neuroendocrine carcinoma (one from each) and undefined tumours (*n*=5). The distribution of TUNEL-staining level types was not statistically different among the various salivary malignancy subgroups.

The 5Y survival rate dropped with larger T, N, M and stage values. For T=1, 5Y survival was 70%, but dropped to 25% for T=3–4 (*P*=0.038). For N=0, 5Y survival was 53% but for any positive N the survival rate dropped to 0 (*P*=0.01). Similarly, for any positive M, survival rate dropped to 0 (*P*=0.16). For stage 1 patients, the 5Y survival rate was 70%, and for patients in advanced stages (3–4) it dropped to 20–26% (*P*=0.05).

Most of the tumours were located in the parotid gland (*n*=38, 55%), whereas in the Sm, Sl and minor salivary glands there were seven, two and 19 cases in each. Of the 19 minor salivary tumours, 12 were located in the palate and seven were located in other sites of the oral mucosa. Post surgical margins were healthy and remote from the tumour by at least 1 cm in all cases. The distribution of the tumours among the various salivary glands was not significantly different, nor was the distribution of TUNEL staining levels in each of the salivary glands. The distribution scale of malignant lesions according to size (T) was up to 2 cm (T1), 2–4 cm (T2), 4–6 cm (T3) and >6 cm (T4), Most of the tumours (67%) were T1–T2 tumours. Although 15% (9/56) had neck metastasis (N1=4, N2=5), only 5% of the patients (3/56) had distant metastasis (positive M) (Table 1).

### Tumour markers

The immunohistochemical analysis demonstrated positive staining for TUNEL in 52% of the salivary tumours analysed. These were composed of 35% moderately stained tumours (level 1) and 17% profoundly stained tumours (level 2). There was no significant difference among the levels of TUNEL staining according to either the specific type of salivary tumour involved or salivary gland involved.

Positive staining for p53 was found in 38% of the tumours (28% were stained with level 1 and 10% with level 2), whereas those stained with Ki67 revealed the following rates: 75% of all tumours for level 1 (1–19%), 17% for level 2 (20–39%) and 8% for level 3 (40–60%). All healthy tissues surrounding the tumours were not stained positively for any of these markers.

The correlation rates between TUNEL and Ki67 was 58% (*P*=0.0001) and between TUNEL and p53 it was 50% (*P*=0.035). The correlation rate between TUNEL and *n* values was rather high (*R*=0.50, *P*=0.0002), (Table 2). The level of TUNEL staining also correlated with the histopathological grading of the tumour (*P*=0.017), (Table 1). As can be seen in Table 3, the 5Y survival frequency dropped significantly to 17% in tumours where TUNEL and Ki67 were concomitantly positively stained (*n*=12, *P*=0.005) and when p53 also stained positively (*n*=3) the 5Y survival frequency dropped to 0.

### TUNEL staining and neck metastasis (N values)

The level of the TUNEL staining grew larger with the N-positive rate (as demonstrated by clinical palpation of the neck), (*P*=0.014). Interestingly, similar high correlation rate was found between TUNEL staining level and positive neck metastasis as defined by the pathological analysis (post-operative) (*P*=0.011), but not as defined by the preoperative CT imaging (*P*=0.54). Moreover, TUNEL-staining level was also found to indicate an extracapsular spread from the neck metastasis (a lethal phenomenon) as can be seen in Table 1.

### TUNEL staining and survival rates

**Figure 1:**
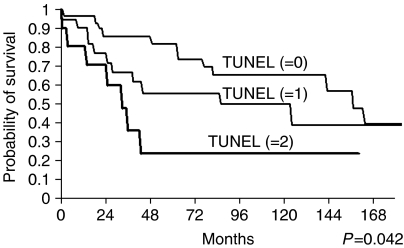
Survival probability curves of salivary-gland tumour patients according to TUNEL immunostaining levels. Kaplan–Meier analysis (age-adjusted) showed poor survival in patients with positive TUNEL expression. Follow-up of 120 months showed 24% survival of TUNEL – level 2-positive patients compared with 66% survival of patients with no detectable TUNEL expression. (*P*=0.042).

**Figure 2:**
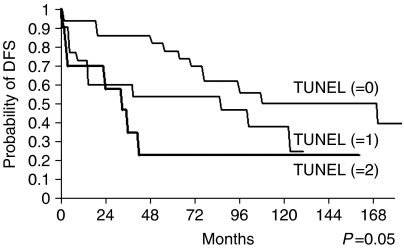
DFS probability curves of salivary-gland tumour patients according to TUNEL expression levels. Kaplan–Meier analysis (age-adjusted) showed significantly poorer DFS in patients with positive TUNEL staining than those with negative (0) TUNEL expression. Follow-up of 120 months (10 years) showed 23% survival of level 2-positive TUNEL-staining patients, compared with 78% survival of patients with negative staining (*P*=0.05).

**Figure 3:**
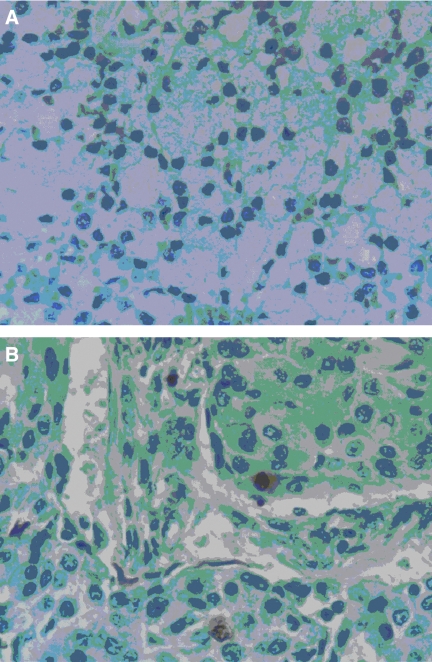
Immunohistochemical staining of TUNEL in patients with salivary malignant tumours. Formalin-fixed, paraffin-embedded  mm sections of salivary gland tumours were subjected to immunostaining of TUNEL. Representative photomicrographs of negative (**A**) acinic cell carcinoma and positive (**B**) mucoepidermoid TUNEL staining are presented.

According to the age-adjusted Kaplan–Meier analysis performed, the survival and DFS probability rates dropped with the TUNEL level of staining (Figures 1, 2 and 3). Thus, although the 5 and 10 years of survival rates for negative TUNEL staining (=0) were 83 and 66%, respectively, these values dropped for positive TUNEL staining. When the staining level=1, they dropped to 57 and 50%, respectively, and when the staining level=2, they both dropped to 24% (*P*=0.042). Similarly, the DFS rates for negative TUNEL staining (=0) at 5 and 10 years were 78 and 50%, respectively, but these, too, dropped for positive TUNEL staining. When the staining level=1, they dropped to 54 and 25%, respectively, and when the staining level=2, they both dropped to 23% (*P*=0.05).

## DISCUSSION

TUNEL was found to be a most powerful marker for predicting poor prognosis in salivary malignancies. Concurrently it correlated with the most important clinical characteristics currently used for prognosis predicting histopathological grade, metastasis spread, extra-capsular spread and stage. Moreover, TUNEL was also found to correlate positively with the staining of both p53 and Ki67, which are considered the most established markers of carcinogenesis and proliferation (in spite of the fact that our currently employed IHC analysis of p53 may not fully reflect the mutational status of this gene). Most interestingly, these clinical and histopathological markers also correlated with each other, giving the results further credence. This is the first time that such findings are concomitantly demonstrated in salivary malignancies. Owing to the well-known diversity among various malignancies in salivary glands it is often impossible to draw significant conclusions from an analysis of a limited number of tumours or from only one type of tumour. Accordingly, we chose to study a relatively large number of salivary tumours (*n*=66) including different types in one cohort, using their associated survival and DFS probabilities as the most important clinical end points. These were correlated with both clinical characteristics and histopathological markers mentioned previously.

Apart form the obvious clinical significance of finding TUNEL to be a powerful predictor for recurrences and poor survival in salivary malignancies, the concomitant positive staining of TUNEL, p53 and Ki67 may shed further light on the carcinogenetic pathogenesis of salivary malignancies.

Our results are supported by previous studies in which the frequency of apoptosis in salivary malignant tumours was reportedly higher than in benign tumours (Nagao *et al*, 1998; Soini *et al*, 1998), and by another study reporting that a higher apoptosis index was assessed by the TUNEL method in the solid pattern of adenoid cystic carcinoma than in the glandular pattern (the solid-pattern carcinoma is known to have a poorer prognosis) (Fujita *et al*, 1999). In this regard, it is interesting to note that previous studies found the level of positive TUNEL staining to be relatively high not only in salivary glands malignancies but also in oral cancer (Loro *et al*, 1999; Nagler *et al*, 1999, 2003). Our results are also supported by multiple recent studies of various non-salivary malignancies which demonstrated that high scores of Ki67 and p53 staining predict a higher tumourogenic grade, a higher rate of metastasis spread and a poorer prognosis, whereas low scores of Ki67 and p53 are associated with prognostically better histopathologic features (Saleh *et al*, 2000; Ben-Izhak *et al*, 2001, 2002, 2003; Jalava *et al*, 2006). Furthermore, in gastric cancer significant relationships between TUNEL, Ki67 and grade have been reported (Jesionek-Kupnicka *et al*, 2002), whereas in oral leukoplakia higher levels of TUNEL, Ki67 and p53 have been reported, indicating increased instability of the genome and higher severity of the dysplasia and the clinical stage (Kovesi and Szende, 2004). Ki67 and p53 were strongly expressed in high-grade prostate cancer tumours (Szabo *et al*, 2000), and higher grade breast carcinoma tumours have been reported as characterised by higher levels of p53 and TUNEL staining (Gonzalez-Campora *et al*, 2000). Bladder cancer grade, p53, Ki67, TUNEL and poor survival have been positively correlated (Shiina *et al*, 1999) and similar results have been found for both non-Hodgkin's lymphoma. (Korkolopoulou *et al*, 1998) and adrenocortical carcinoma (Khoury *et al*, 2003).

We currently employed TUNEL staining in salivary tumours for evaluation of their malignant profile as TUNEL examination has often been performed by investigators in other cancer types and in view of the fact that an unbalanced admixture of proliferative cells and apoptotic cells in neoplastic parenchyma is related to tumour growth. When the growth features of tumours are discussed, not only cell proliferation but also cell death, especially apoptosis, should be taken into consideration. However, TUNEL *in situ* technique for the detection of apoptosis is not completely specific, as overlap between apoptotic and necrotic cell death has been reported, which may result in the fact that some of the apoptotic cells do not stain (Finlay *et al*, 1988; Wyllie *et al*, 1999). TUNEL evaluation and scoring is a difficult task for clinical application. Early DNA fragmentation may not be readily detected by the TUNEL assay and this may lead to underestimation of the apoptotic values. Moreover, the margin of error in scoring TUNEL between cases is too narrow to allow for definitive categorization. The report of Grasl-Kraupp *et al* (1995), which found that TUNEL failed to distinguish between apoptotic and necrotic cells, strengthens this point. Thus, the exact biological pathway expressed by the positive TUNEL staining is yet to be fully elucidated and its exploration is highly warranted. This may lead to finding a cellular target (associated or non-associated with p53 and/or Ki67), which might be used for anti-carcinogenetic therapy. Finally, it is noteworthy to mention a paper published recently by Jalava *et al* (2006), which seems to show that mitotic activity and apoptotic activity are related in breast cancer and that Ki-67 may not be the best possible proliferation-associated prognosticator but rather the mitotic count. Furthermore, in 2000 this group (Jalava *et al*, 2000) showed that Bcl-2 immunostaining could potentially replace TUNEL as its prognostic value is well-established in breast cancer.

In summary, the demonstrated significant concurrent staining of salivary malignant tumours for TUNEL, p53 and Ki67 indicates a biological role of all three markers in the pathogenesis of salivary malignancies. The significant correlation between TUNEL staining and the survival and DFS rates and the histopathological grade, stage and metastasis spread of these malignancies is of paramount importance for both prognostic as well as follow-up purposes. Further analysis of these markers role in the pathogenesis of specific salivary malignancies is warranted in the future, being based on larger cohorts of the tumours.

## Figures and Tables

**Table 1:** Distribution of TUNEL levels by grade, T, N and extracapsular spread

**TUNEL level**	**0 patients (%)**	**One patient (%)**	**Two patients (%)**
*(A) Grade level*			
Low (*n*=10)	8 (62)	2 (17)	
Intermediate (*n*=8)	2 (15)	4 (33)	2 (22)
High (*n*=16)	3 (23)	6 (50)	7 (78)
Total (*n*=34)	13 (100)	12 (100)	9 (100)
			
*(B) T level*			
1 (*n*=20)	12 (41)	7 (41)	1 (10)
2 (*n*=24)	12 (41)	7 (41)	5 (50)
3 (*n*=4)	1 (4)		3 (30)
4 (*n*=8)	4 (14)	3 (18)	1 (10)
Total (*n*=56)	29 (100)	17 (100)	27 (100)
			
*(C) N level*			
0 (*n*=47)	28 (97)	13 (76)	6 (60)
1 (*n*=4)	1 (3)	2 (12)	1 (10)
2 (*n*=5)		2 (12)	3 (30)
Total (*n*=56)	29 (100)	17 (100)	10 (100)
			
*(D) Neck extracapsular spread*			
No	13 (100)	4 (80)	4 (80)
Yes	—	1 (20)	1 (20)
Total (*n*=23)	13 (100)	5 (100)	5 (100)

N=neck metastasis; T=size; TUNEL=terminal deoxynucleotidyl transferase-mediated biotinylated deoxyuridine-triphosphate biotin nick-end labelling.

Exact test: *P*=0.017 significant.

Exact test: *P*=0.14.

Exact test: *P*=0.014.

*P*=0.05.

**Table 2:** Correlation factor between TUNEL level of staining and T, N, M and stage values

**Correlation factor between**	**TUNEL**
*Stage*	*r*=0.25
	(*P*=0.08)
T	*r*=0.16
	(*P*=0.23)
N	*r*=0.50
	(*P*=0.0002)
M	*r*=0.12
	(*P*=0.40)

M=distant metastasis; N=neck metastasis; T=size; TUNEL=terminal deoxynucleotidyl transferase-mediated biotinylated deoxyuridine-triphosphate biotin nick-end labelling.

**Table 3:** 5Y survival frequency by concomitant TUNEL, Ki67 and p53 staining levels (0 staining depicts negative staining for all marker/s analysed, >0 staining depicts positive staining for all marker/s analysed)

**(A)** **TUNEL and Ki67**	**5Y survival patients**	**5Y survival patients (%)**
0 (*n*=28)	19/28	68
>0 (*n*=12)	2/12	17
Total (*n*=40)	21/40	
		
**(B)** **TUNEL, Ki67 and p53**		
0 (*n*=10)	10/10	100
>0 (*n*=3)	0/3	0
Total (*n*=13)	10/13	

TUNEL=terminal deoxynucleotidyl transferase-mediated biotinylated deoxyuridine-triphosphate biotin nick-end labelling; 5Y=5 year.

Exact test: *P*=0.005 significant.

Exact test: *P*=0.003 significant.
